# Membrane Sealant Poloxamer P188 Protects Against Isoproterenol Induced Cardiomyopathy in Dystrophin Deficient Mice

**DOI:** 10.1186/1471-2261-11-20

**Published:** 2011-05-16

**Authors:** Christopher F Spurney, Alfredo D Guerron, Qing Yu, Arpana Sali, Jack H van der Meulen, Eric P Hoffman, Kanneboyina Nagaraju

**Affiliations:** 1 Children's National Heart Institute, Division of Cardiology, Children's National Medical Center, Washington, DC USA; 2 Department of Integrative Systems Biology, George Washington University School of Medicine and Public Health, Washington, DC USA; 3 Center for Genetic Medicine Research, Children's National Medical Center, Washington, DC USA

**Keywords:** Duchenne muscular dystrophy, mdx, poloxamer, cardiomyopathy, isoproterenol

## Abstract

**Background:**

Cardiomyopathy in Duchenne muscular dystrophy (DMD) is an increasing cause of death in patients. The absence of dystrophin leads to loss of membrane integrity, cell death and fibrosis in cardiac muscle. Treatment of cardiomyocyte membrane instability could help prevent cardiomyopathy.

**Methods:**

Three month old female mdx mice were exposed to the β_1 _receptor agonist isoproterenol subcutaneously and treated with the non-ionic tri-block copolymer Poloxamer P188 (P188) (460 mg/kg/dose i.p. daily). Cardiac function was assessed using high frequency echocardiography. Tissue was evaluated with Evans Blue Dye (EBD) and picrosirius red staining.

**Results:**

BL10 control mice tolerated 30 mg/kg/day of isoproterenol for 4 weeks while death occurred in mdx mice at 30, 15, 10, 5 and 1 mg/kg/day within 24 hours. Mdx mice tolerated a low dose of 0.5 mg/kg/day. Isoproterenol exposed mdx mice showed significantly increased heart rates (p < 0.02) and cardiac fibrosis (p < 0.01) over 4 weeks compared to unexposed controls. P188 treatment of mdx mice significantly increased heart rate (median 593 vs. 667 bpm; p < 0.001) after 2 weeks and prevented a decrease in cardiac function in isoproterenol exposed mice (Shortening Fraction = 46 ± 6% vs. 35 ± 6%; p = 0.007) after 4 weeks. P188 treated mdx mice did not show significant differences in cardiac fibrosis, but demonstrated significantly increased EBD positive fibers.

**Conclusions:**

This model suggests that chronic intermittent intraperitoneal P188 treatment can prevent isoproterenol induced cardiomyopathy in dystrophin deficient mdx mice.

## Background

Since cardiomyopathy is an increasing cause of morbidity and mortality in Duchenne muscular dystrophy (DMD) patients, pre-clinical drug trials in mice are an important initial step to investigate potential therapeutic pathways for the treatment of DMD cardiomyopathy [1]. However, previous studies in mdx mice show that the cardiomyopathy develops slowly and only becomes evident by non-invasive echocardiography around 9 months of age [2,3]. Mdx mice are asymptomatic from a cardiac standpoint at this age and show no signs of heart failure throughout their life. This mild phenotype can make it difficult to measure significant cardiac endpoints for pre-clinical drug studies and requires 6 to 12 month long treatment duration, making these trials very expensive, time consuming and cumbersome [4].

In an attempt to hasten the phenotype, many investigators use short treatment courses with invasive models of cardiac stress including cardiac cannulation with dobutamine or isoproterenol infusion and aortic banding [5-10]. These studies confirm that untreated mdx mice do not tolerate cardiac stress normally due to the lack of dystrophin in cardiac muscle. This inability to handle stress is thought to be related to membrane instability from the loss of dystrophin. Experiments by Yasuda et al. (2005) showed that intact, isolated dystrophin-deficient cardiac myocytes have reduced compliance and increased susceptibility to stretch-mediated calcium overload, leading to cell contracture and death [9].

Improvement of membrane stability is one potential therapeutic pathway in DMD. Poloxamer 188 (P188) is known to stabilize red blood cell membranes in sickle cell disease [11]. P188 is a non-ionic triblock co-polymer, poly(ethylene oxide)_80_-poly(propylene oxide)_27_-poly(ethylene oxide)_80_, shown to insert into artificial lipid monolayers and repair damaged biological membranes. Based on these properties, Yasuda et al. (2005) studied P188 in the dystrophin deficient mdx mouse heart using an open chest protocol with a catheter in the left ventricle. This model showed that administration of P188 during dobutamine infusion prevented the development of acute cardiac failure [9].

In this study, we use chronic, intermittent dosing of P188 with a continuous, low dose isoproterenol cardiac stress model and non-invasive assessment of cardiac function. Mdx mice only tolerated significantly reduced isoproterenol concentrations compared to wild type mice and developed decreased shortening fraction and increased cardiac fibrosis over 4 weeks. Treatment with P188 may prevent the decrease in shortening fraction during isoproterenol exposure compared to untreated mdx mice.

Current therapies for cardiomyopathy in DMD are non-specific, but P188 directly targets membrane instability which is known to be one of the major pathological defects in dystrophin deficient cells. These experiments show that chronic, intermittent P188 dosing may provide a potential direct therapy for cardiomyopathy in Duchenne muscular dystrophy.

## Methods

### Animal care

The investigation conforms to the Guide for the Care and Use of Laboratory Animals published by the US National Institutes of Health (NIH Publication No. 85-23, revised 1985). All mice were handled according to the Institutional Animal Care and Use Committee guidelines (protocol #01002). Generally, 2-3 month old female C57BL/10ScSn-Dmd*^mdx^*/J (*mdx*) and C57BL/10ScSn (wild type) mice weighing 20-25 grams were purchased from the Jackson Laboratory (Bar Harbor, Ma). All mice were housed in an individually vented cage system with a 12 hour light-dark cycle and received standard mouse chow and purified water *ad libitum*.

### Drug treatment

Subcutaneous pumps containing isoproterenol (Sigma, St. Louis, Mo) diluted in normal saline at doses of 30, 15, 10, 5, 1 or 0.5 mg/kg/day were implanted in mice. Mice also received daily intraperitoneal injections of either normal saline (0.2 cc) or 460 mg/kg/dose of P188 (BASF Corporation, Florham Park, NJ) in normal saline (0.2 cc) [12]. Two separate trials of 2 and 4 weeks duration were performed. Mice received the initial injection prior to osmotic pump implantation. Mice were injected in the morning except during echocardiography measurements when injections were performed immediately after imaging.

### Subcutaneous pump insertion

The Alzet 2004 osmotic pump (Durect Corporation, Cupertino, Ca) (200 microliter reservoir with a delivery rate of 0.25 microliters/hour) was filled with the appropriate isoproterenol solution and allowed to equilibrate for approximately 40 hours in normal saline per manufacturer's recommendations prior to implantation. Mice were anesthetized using approximately 3% inhaled isoflurane delivered via a nose cone with a passive exhaust system. A small area on the back of the mouse, slightly posterior to the scapulae, was shaved and surgically prepped using betadine and alcohol solutions. A small 1-2 cm incision was made in the midscapular region. A hemostat was inserted and gently opened and closed to create a pocket for the pump, approximately 1 cm longer and wider than the pump to allow for some free movement. An osmotic pump was inserted with the exit port entering the pocket first. The incision was closed with 2-3 sutures.

### Echocardiography

Mice were anesthetized with 1-2% isoflurane in 100% oxygen and scanning was performed over 20 minutes using a high frequency ultrasound probe (RMZ 702a, Vevo 660, VisualSonics, Toronto, Canada) as previously described [3]. Qualitative and quantitative measurements were made offline using analytic software (VisualSonics, Toronto, Canada). A blood pressure (BP) cuff was placed around the tail and the tail was then placed in a sensor assembly for non-invasive blood pressure monitoring (SC1000, Hatteras Instruments, Cary, NC). Ten consecutive blood pressure measurements were obtained and the average pressure used for each mouse.

### In vitro skeletal muscle force contraction

Experiments were conducted on the extensor digitorum longus (EDL) muscle of the right hindlimb of mice as previously described [13]. The muscle length was measured with calipers and the optimal fiber length calculated by multiplying the optimal muscle length by 0.45, the established fiber length/muscle length ratio for EDL muscle [14]. The muscle mass was weighed after removal of the muscle from the bath. The muscle specific force, a measure of intrinsic force generation of muscle, was calculated using the maximal isometric force, the muscle mass and the fiber length according the following equation: specific force = maximal isometric force/[muscle mass × (density of muscle tissue * fiber length)^-1^]. Muscle tissue density used was 1.056 kg/L.

### Collagen quantification

Five paraffin sections of cardiac tissue (7 um thickness) were stained with picrosirius red (Histoserv, Gaithersburg, Md). The tissue was magnified under a light microscope at an objective of 4X and a digital image of the entire section was obtained using computer software (Olympus C.A.S.T. Stereology System, Olympus America Inc., Center Valley, PA). These digital images were processed using Image J (NIH) with additional threshold color plug-ins to process jpeg images. Pixels corresponding to the area stained in red in the previously described technique were normalized to the total pixel area of the tissue image and the results were expressed as percent of collagen. Images were analyzed by two independent, blinded investigators.

### Evans Blue Dye (EBD) quantification

Three mice per group received an intra-peritoneal injection of 1% EBD in normal saline (Sigma, St. Louis, Mo) at a dose of 100 microliters per 10 grams body weight. Less than 18 hours after injection, the mice were sacrificed and the heart tissue snap-frozen in isopentane cooled with liquid nitrogen and stored at -80°C. The tissue was cut into 7 micrometer thick sections and incubated in ice-cold acetone at -20°C for 3 minutes and mounted using Vectashield (Vector Laboratories, Burlingame, CA). Slides were examined under a Nikon Eclipse fluorescent microscope using Texas Red filter and fibers showing red fluorescence were counted over the entire heart tissue slice by two independent, blinded investigators and expressed as absolute number of EBD positive fibers per heart.

### Statistical analysis

At each time point, normality was tested for each measurement using the Shipiro-Wilk normality test. Those having a normal distribution were analyzed using a parametric t-test (Table 1) or an one way ANOVA with adjustment of pair-wise post hoc tests for multiple comparisons using the Sidak method (Tables 2 and 3). For those measurements not conforming to normality, non-parametric tests were used. At time 0, comparisons between BL10 and MDX untreated mice used a Wilcoxon rank sum test to compare medians (Table 4). At time 2 and 4, comparisons between all 5 treatment groups used a Kruskal-Wallis test to compare medians (Tables 5 and 6). For comparisons with a significant overall p-value from the Kruskal-Wallis test, individual post-hoc pair-wise comparisons were done using a Wilcoxon rank sum test. All p-values from post-hoc tests were adjusted for multiple comparisons using the Sidak method. P-values presented in Tables 5 and 6 are Sidak adjusted p-values. Specific muscle force testing was analyzed using a one-way ANOVA. Percent collagen was analyzed using Kruskal-Wallis test with Dunn's post-hoc comparisons. EBD counts were analyzed using an unpaired t-test. All analyses used a critical p-value of 0.05 to assign significance.

**Table 1:** Baseline parametric comparisons of cardiac parameters in untreated 10 week old BL10 and mdx mice.

		BL10		MDX	
**Measurement**	**N**	**Mean ± SD**	**N**	**Mean ± SD**	**P-value**

Heart rate (bpm)	5	473 ± 35	22	458 ± 48	0.5370

SF%	10	30.91 ± 1.64	9	32.60 ± 3.08	0.1471

EF%	10	56.37 ± 3.65	9	58.38 ± 2.29	0.1739

LVID (d) (mm)	10	4.00 ± 0.16	9	4.05 ± 0.19	0.5776

LVPW (d) (mm)	10	0.54 ± 0.06	9	0.56 ± 0.03	0.4305

LV mass (mg)	10	77.92 ± 8.36	9	72.33 ± 4.67	0.0945

AoV vel (mm/s)	5	1079 ± 138	9	969 ± 121	0.1439

Systolic BP (mmHg)	5	89 ± 6	22	73 ± 6	<0.0001

Bpm - beats per minute; SF% - shortening fraction percent; EF% - ejection fraction percent; LVID(d) - left ventricular internal diameter in diastole; LVPW(d) - left ventricular posterior wall thickness in diastole; LV - left ventricle; AoV vel - aortic valve velocity; BP - blood pressure; mmHg - millimeters of mercury; SD - standard deviation; mm - millimeters; mm/s - millimeters per second; mg - milligrams

**Table 2:** Parametric comparisons of cardiac parameters after 2 weeks of exposure to isoproterenol and treatment with P188 in BL10 and mdx mice.

Measurement	Group	N	Mean ± SD	Overall p-value	Significantly different groups
SF%	BL10 - Untx	3	31.47 ± 0.56		BL10-Untx vs. BL10-Iso (p < 0.001)
			
	BL10 - Iso	7	49.33 ± 3.87	<0.0001	BL10-Untx vs. MDX-Iso+P188 (p = 0.001) BL10-Iso vs. MDX-Untx (p < 0.001)
			
	MDX - Untx	6	30.31 ± 2.50		BL10-Iso vs. MDX-Iso (p < 0.001)
			
	MDX - Iso	10	38.06 ± 4.81		BL10-Iso vs. MDX-Iso+P188 (p = 0.019)
			
	MDX - Iso + P188	9	42.95 ± 3.27		MDX-Untx vs. MDX-Iso (p = 0.003) MDX-Untx vs. MDX-Iso+P188 (p < 0.001)

LVID (d) (mm)	BL10 - Untx	3	4.07 ± 0.35		
			
	BL10 - Iso	7	3.70 ± 0.14		
			
	MDX - Untx	6	3.84 ± 0.27	0.0011	BL10-Untx vs. MDX-Iso+P188 (p = 0.007) MDX-Untx vs. MDX-Iso+P188 (p = 0.030)
			
	MDX - Iso	10	3.42 ± 0.37		
			
	MDX - Iso + P188	9	3.37 ± 0.22		

LVPW (d) (mm)	BL10 - Untx	3	0.56 ± 0.01		BL10-Untx vs. BL10-Iso (p = 0.001)
			
	BL10 - Iso	7	0.85 ± 0.07		BL10-Untx vs. MDX-Iso (p = 0.001)
			
	MDX - Untx	6	0.60 ± 0.09	<0.0001	BL10-Untx vs. MDX-Iso+P188 (p < 0.001) BL10-Iso vs. MDX-Untx (p = 0.001)
			
	MDX - Iso	10	0.88 ± 0.14		MDX-Untx vs. MDX-Iso (p < 0.001)
			
	MDX - Iso + P188	9	0.94 ± 0.07		MDX-Untx vs. MDX-Iso+P188 (p < 0.001)

LV mass (mg)	BL10 - Untx	3	77.48 ± 1.60		BL10-Untx vs. BL10-Iso (p = 0.002)
			
	BL10 - Iso	7	105.7 ± 12.22		BL10-Iso vs. MDX-Untx (p < 0.001)
			
	MDX - Untx	6	73.16 ± 9.55	<0.0001	BL10-Iso vs. MDX-Iso (p < 0.001)
			
	MDX - Iso	10	81.84 ± 9.62		BL10-Iso vs. MDX-Iso+P188 (p = 0.003
			
	MDX - Iso + P188	9	85.26 ± 9.21		

AoV vel (mm/s)	BL10 - Untx	3	1107 ± 49		
			
	BL10 - Iso	7	1194 ± 152		BL10-Iso vs. MDX-Iso (p = 0.003)
			
	MDX - Untx	6	943 ± 67	<0.0001	MDX-Untx vs. MDX-Iso+P188 (p = 0.001)
			
	MDX - Iso	10	885 ± 195		MDX-Iso vs. MDX-Iso+P188 (p < 0.001)
			
	MDX - Iso + P188	9	1317 ± 150		

Systolic BP (mmHg)	BL10 - Untx	3	92 ± 6		
			
	BL10 - Iso	7	107 ± 5		BL10-Iso vs. MDX-Untx (p = 0.023)
			
	MDX - Untx	3	88 ± 3	0.0005	BL10-Iso vs. MDX-Iso (p < 0.001)
			
	MDX - Iso	5	82 ± 5		BL10-Iso vs. MDX-Iso+P188 (p = 0.031)
			
	MDX - Iso + P188	6	92 ± 13		

SF% - shortening fraction percent; LVID(d) - left ventricular internal diameter in diastole; LVPW(d) - left ventricular posterior wall thickness in diastole; LV - left ventricle; AoV vel - aortic valve velocity; BP - blood pressure; mmHg - millimeters of mercury; SD - standard deviation; mm - millimeters; mm/s - millimeters per second; mg - milligrams; iso - isoproterenol; untx - untreated

**Table 3:** Parametric comparisons of cardiac parameters after 4 weeks of exposure to isoproterenol and treatment with P188 in BL10 and mdx mice.

Measurement	Group	N	Mean ± SD	Overall p-value	Significantly different groups
SF%	BL10 - Untx	3	33.34 ± 1.15		BL10-Untx vs. BL10-Iso (p = 0.019) BL10-Untx vs. MDX-Iso+P188 (p = 0.017)
			
	BL10 - Iso	7	45.13 ± 4.71		BL10-Iso vs. MDX-Untx (p < 0.001)
			
	MDX - Untx	6	29.64 ± 3.11	<0.0001	BL10-Iso vs. MDX-Iso (p = 0.006)
			
	MDX - Iso	9	35.37 ± 5.77		MDX-Untx vs. MDX-Iso+P188 (p < 0.001)
			
	MDX - Iso + P188	5	45.97 ± 6.23		MDX-Iso vs. MDX-Iso+P188 (p = 0.007)

EF%	BL10 - Untx	3	59.15 ± 1.89		
			
	BL10 - Iso	7	69.72 ± 5.56		
			
	MDX - Untx	6	57.25 ± 7.78	0.0027	BL10-Iso vs. MDX-Untx (p = 0.020)
			
	MDX - Iso	9	57.84 ± 4.56		BL10-Iso vs. MDX-Iso (p = 0.013)
			
	MDX - Iso + P188	5	68.28 ± 9.89		

LVID (d) (mm)	BL10 - Untx	3	4.03 ± 0.12		
			
	BL10 - Iso	7	3.73 ± 0.13		BL10-Untx vs. MDX-Iso+P188 (p = 0.002)
			
	MDX - Untx	6	4.02 ± 0.07	0.0001	BL10-Iso vs. MDX-Iso (p = 0.029)
			
	MDX - Iso	9	3.54 ± 0.34		MDX-Untx vs. MDX-Iso (p = 0.0125)
			
	MDX - Iso + P188	5	3.27 ± 0.32		MDX-Untx vs. MDX-Iso+P188 (p < 0.001)

LVPW (d) (mm)	BL10 - Untx	3	0.62 ± 0.10		
			
	BL10 - Iso	7	0.80 ± 0.08		BL10-Untx vs. BL10-Iso (p = 0.022)
			
	MDX - Untx	6	0.59 ± 0.01	<0.0001	BL10-Untx vs. MDX-Iso (p = 0.001) BL10-Untx vs. MDX-Iso+P188 (p = 0.004)
			
	MDX - Iso	9	0.86 ± 0.09		BL10-Iso vs. MDX-Untx (p < 0.001)
			
	MDX - Iso + P188	5	0.84 ± 0.07		MDX-Untx vs. MDX-Iso (p < 0.001) MDX-Untx vs. MDX-Iso+P188 (p < 0.001)

LV mass (mg)	BL10 - Untx	3	75.16 ± 7.16		
			
	BL10 - Iso	7	98.33 ± 11.06		
			
	MDX - Untx	6	76.88 ± 3.36	0.0019	BL10-Untx vs. BL10-Iso (p = 0.012)
			
	MDX - Iso	9	87.22 ± 11.08		BL10-Iso vs. MDX-Untx (p = 0.003)
			
	MDX - Iso + P188	5	89.15 ± 7.54		

AoV vel (mm/s)	BL10 - Untx	3	1004 ± 87		
			
	BL10 - Iso	7	1077 ± 186		
			
	MDX - Untx	6	1105 ± 117	0.0388	None
			
	MDX - Iso	9	1274 ± 159		
			
	MDX - Iso + P188	5	1253 ± 183		

Systolic BP (mmHg)	BL10 - Untx	3	94 ± 5		
			
	BL10 - Iso	7	99 ± 8		BL10-Untx vs. MDX-Iso (p = 0.011)
			
	MDX - Untx	3	87 ± 8	0.0001	BL10-Iso vs. MDX-Iso (p < 0.001)
			
	MDX - Iso	9	73 ± 11		BL10-Iso vs. MDX-Iso+P188 (p = 0.006)
			
	MDX - Iso + P188	4	77 ± 6		

Diastolic BP (mmHg)	BL10 - Untx	3	50 ± 9		
			
	BL10 - Iso	7	85 ± 8		
			
	MDX - Untx	3	67 ± 3	<0.0001	BL10-Untx vs. BL10-Iso (p < 0.001) BL10-Iso vs. MDX-Untx (p = 0.022)
			
	MDX - Iso	9	61 ± 8		BL10-Iso vs. MDX-Iso (p < 0.001)
			
	MDX - Iso + P188	4	58 ± 3		BL10-Iso vs. MDX-Iso+P188 (p < 0.001)

Mean BP (mmHg)	BL10 - Untx	3	64 ± 7		
			
	BL10 - Iso	7	89 ± 7		BL10-Untx vs. BL10-Iso (p < 0.001)
			
	MDX - Untx	3	73 ± 4	<0.0001	BL10-Iso vs. MDX-Untx (p = 0.018)
			
	MDX - Iso	9	63 ± 7		BL10-Iso vs. MDX-Iso (p < 0.001)
			
	MDX - Iso + P188	4	64 ± 2		BL10-Iso vs. MDX-Iso+P188 (p < 0.001)

SF% - shortening fraction percent; EF% - ejection fraction percent; LVID(d) - left ventricular internal diameter in diastole; LVPW(d) - left ventricular posterior wall thickness in diastole; LV - left ventricle; AoV vel - aortic valve velocity; BP - blood pressure; mmHg - millimeters of mercury; SD - standard deviation; mm - millimeters; mm/s - millimeters per second; mg - milligrams; iso - isoproterenol; untx - untreated

**Table 4:** Baseline non-parametric comparisons in untreated 10 week old BL10 and mdx mice.

		BL10			MDX		
**Measurement**	**N**	**Median**	**Range**	**N**	**Median**	**Range**	**P-value**

Diastolic BP (mmHg)	5	50	41 - 69	22	42	32 - 69	0.0650

Mean BP (mmHg)	5	63	57 - 76	22	51	44 - 76	0.0044

BP - blood pressure; mmHg - millimeters of mercury

**Table 5:** Non-parametric comparisons of cardiac parameters after 2 weeks of exposure to isoproterenol and treatment with P188 in BL10 and mdx mice.

Measurement	Group	N	Median	Range	Overall p-value	Significantly different groups
Heart rate (bpm)	BL10 - Untx	3	451	410 - 466		
			
	BL10 - Iso	7	645	593 - 691		BL10-Iso vs. MDX-Untx (p = 0.0267)
			
	MDX - Untx	6	454	416 - 477	0.0001	MDX-Untx vs. MDX-Iso (p = 0.0325)
			
	MDX - Iso	10	593	462 - 632		MDX-Untx vs. MDX-Iso+P188 (p = 0.0129)
			
	MDX - Iso + P188	9	667	632 - 686		MDX-Iso vs. MDX-Iso+P188 (p = 0.0008)

EF%	BL10 - Untx	3	60.35	59.17 - 61.32		
			
	BL10 - Iso	7	80.03	77.40 - 86.59		BL10-Iso vs. MDX-Untx (p = 0.0267)
			
	MDX - Untx	6	59.00	50.93 - 62.22	0.0001	BL10-Iso vs. MDX-Iso (p = 0.0060)
			
	MDX - Iso	10	65.00	50.15 - 75.10		MDX-Untx vs. MDX-Iso+P188 (p = 0.0149)
			
	MDX - Iso + P188	9	74.85	69.98 - 81.18		

Diastolic BP (mmHg)	BL10 - Untx	3	74	57 - 86		
			
	BL10 - Iso	7	95	83 - 102		
			
	MDX - Untx	3	58	39 - 62	0.0026	BL10-Iso vs. MDX-Iso (p = 0.0441) BL10-Iso vs. MDX-Iso+P188 (p = 0.0267)
			
	MDX - Iso	5	56	50 - 66		
			
	MDX - Iso + P188	6	53	44 - 60		

Mean BP (mmHg)	BL10 - Untx	3	78	67 - 89		
			
	BL10 - Iso	7	97	89 - 106		
			
	MDX - Untx	3	67	56 - 71	0.0031	BL10-Iso vs. MDX-Iso (p = 0.0431) BL10-Iso vs. MDX-Iso+P188 (p = 0.0267)
			
	MDX - Iso	5	63	59 - 73		
			
	MDX - Iso + P188	6	66	57 - 71		

Bpm - beats per minute; EF% - ejection fraction percent; BP - blood pressure; mmHg - millimeters of mercury; SD - standard deviation; iso - isoproterenol; untx - untreated

**Table 6:** Non-parametric comparisons of cardiac parameters after 4 weeks of exposure to isoproterenol and treatment with P188 in BL10 and mdx mice.

Measurement	Group	N	Median	Range	Overall p-value	Significantly different groups
Heart rate (bpm)	BL10 - Untx	3	450	421 - 476		
			
	BL10 - Iso	7	615	532 - 656		
			
	MDX - Untx	6	438	400 - 489	0.0004	BL10-Iso vs. MDX-Untx (p = 0.0267)
			
	MDX - Iso	9	585	541 - 667		MDX-Untx vs. MDX-Iso (p = 0.0149)
			
	MDX - Iso + P188	5	632	615 - 667		

Bpm - beats per minute; iso - isoproterenol; untx - untreated

## Results

### Isoproterenol cardiac stress model in mdx mice

Using a standard dose of isoproterenol (30 mg/kg/day) known to induce hypertrophy in normal mouse hearts, all mdx mice died within 12 hours [15-18]. Normal BL10 mice tolerated this dose for 4 weeks. Decreasing doses of 15, 10, 5 and 1 mg/kg/day continued to cause 100% mortality in mdx mice within 24 hours. Treatment with P188 did not prevent mdx mice from dying at doses greater than 0.5 mg/kg/day. Therefore, all experiments were performed at a dose of 0.5 mg/kg/day. At this dose, 19/20 mice tolerated treatment for two weeks and 14/15 mice tolerated treatment for 4 weeks. Of the mice that died, one was in the P188 treated, isoproterenol exposed group and one was in the isoproterenol only group. No mice completing the study were excluded from the results when data was reliably obtained.

### Body weights

All the mdx mice started the trial with similar average weights (g): Isoproterenol exposed and P188 treated 21.3 ± 1.3; isoproterenol exposed and saline treated 21.2 ± 1.9; control (saline exposed and saline treated) 20.5 ± 1.5. There were no significant differences in body weights at the end of 4 weeks of treatment between different groups: isoproterenol exposed and P188 treated 24.8 ± 0.9; isoproterenol exposed and saline treated 24.3 ± 1.4; saline exposed and saline treated 23.8 ± 1.86.

### Cardiovascular function

Results are presented in Tables 1, 2, 3, 4, 5 and 6. Baseline data was performed prior to osmotic pump implantation.

### Heart rate

There was no significant difference between heart rates of control (saline exposure and saline treatment) BL10 and mdx mice at baseline, 2 weeks or 4 weeks. After 2 weeks of exposure, isoproterenol significantly increased the hearts rates in mdx mice (p = 0.03) compared to untreated controls. Mdx mice exposed to isoproterenol and treated with P188 also had significantly increased heart rates compared to mdx mice exposed to isoproterenol only (p = 0.001). After 4 weeks of exposure, isoproterenol treated mdx mice had significantly increased heart rates compared to untreated controls (p < 0.02).

### Systolic function

**Figure 1:**
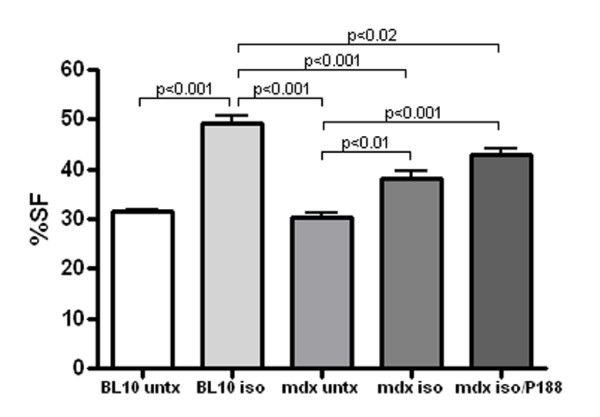
**P188 treated, isoproterenol exposed and isoproterenol exposed mdx mice show significantly increased percent shortening fraction (%SF) compared to untreated mdx controls after 2 weeks of therapy**.

**Figure 2:**
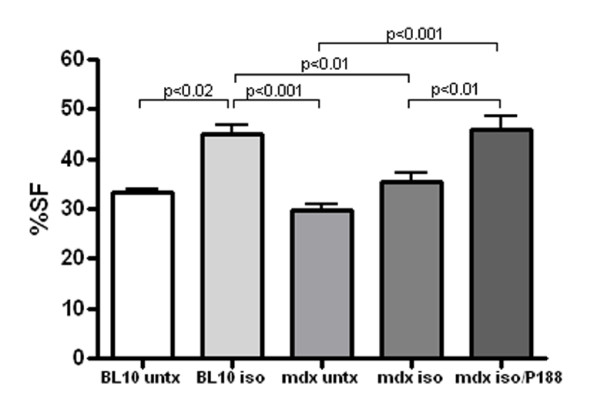
**P188 treated, isoproterenol exposed mdx mice show significantly increased percent shortening fraction (%SF) compared to isoproterenol exposed mdx mice and untreated controls after 4 weeks of therapy**.

There was no significant difference between control BL10 and control mdx mice in shortening fraction (SF) or ejection fraction (EF) at baseline, 2 weeks or 4 weeks. Isoproterenol exposure significantly increased systolic function measured via SF in BL10 mice at 2 weeks (p < 0.001) and 4 weeks (p = 0.02) compared to strain matched untreated control mice. Mdx mice exposed to isoproterenol showed significantly increased SF at 2 weeks compared to untreated mdx controls (p = 0.03). (Figure 1) P188 treatment significantly improved SF compared to untreated, isoproterenol exposed mdx mice at 4 weeks (p = 0.007). (Figure 2) At 2 weeks, independent 2-D measures of EF showed significant increases in P188 treated, isoproterenol exposed mdx mice compared to isoproterenol exposed mdx mice (p < 0.001). At 4 weeks, EF agreed with SF measures and showed no difference between control and isoproterenol treated mdx mice.

### Left ventricular dimensions and mass

There were no significant differences in left ventricular dimensions or mass between control BL10 and mdx mice at baseline, 2 and 4 weeks. The left ventricular internal diameter measured in diastole [LVID(d)] significantly decreased in P188 treated, isoproterenol exposed mdx mice at 2 weeks compared to untreated mdx controls (p = 0.03). At 4 weeks, LVID(d) was also significantly decreased in isoproterenol exposed mdx mice (p < 0.02) and in P188 treated, isoproterenol exposed mdx mice (p < 0.001) compared to untreated mdx controls. Isoproterenol exposure led to an increase in the dimension of the left ventricular posterior wall in diastole [LVPW(d)] in BL10 and mdx mice at 2 and 4 weeks of treatment (p < 0.03) compared to untreated strain matched controls. P188 treatment did not significantly affect LVPW(d). This increase in wall thickness was correlated with 2-D left ventricular mass (LV mass) measures. LV mass was significantly increased after 2 and 4 weeks of isoproterenol exposure in BL10 mice only (p < 0.02).

### Aortic valve maximum velocity

Aortic valve peak velocity did not significantly differ between control BL10 and mdx mice at baseline, 2 and 4 weeks. At 2 weeks of isoproterenol exposure, aortic velocity was significantly increased in mdx mice treated with P188 compared to untreated isoproterenol exposed mdx mice (p < 0.001), but this difference was not seen after 4 weeks of treatment when the two groups had similar velocities.

### Blood pressure

Mdx mice showed significantly decreased systolic and mean blood pressures at baseline compared to BL10 mice (p < 0.005). Mdx mice did not show a significant increase in blood pressure after isoproterenol exposure compared to unexposed controls. P188 treatment did not significant affect blood pressure measurements.

### In vitro skeletal muscle strength

(Additional file 1) After two weeks of exposure to isoproterenol in the mdx mouse (n = 5), the specific force (sPo) was 151 ± 25 kN/m^2 ^in the EDL. In mdx mice exposed to isoproterenol and treated with P188 (n = 5), the sPo was 176 ± 12 kN/m^2 ^and not significantly different. There was also no significant difference after 4 weeks. Mdx mice exposed to isoproterenol (n = 5) had a sPo of 201 ± 36 kN/m^2 ^and mdx mice exposed to isoproterenol and treated with P188 had a sPo 185 ± 25 of kN/m^2 ^(p = 0.5). Mdx mice unexposed to isoproterenol/P188 (n = 3) had a sPo of 167 ± 14 of kN/m^2 ^and did not differ significantly from the other groups.

### Percent cardiac collagen content

**Figure 3:**
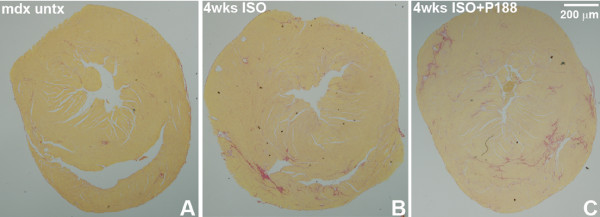
**Representative cross-sectional tissue slices of hearts stained with picrosirius red show minimal fibrosis in untreated mdx hearts (mean 1% collagen; Panel A) and significantly increased fibrosis in mdx hearts exposed to 0.5 mg/kg/day isoproterenol for 4 weeks (mean 3.0% collagen; Panel B) and in mdx hearts exposed to 0.5 mg/kg/day isoproterenol and treated with P188 for 4 weeks (mean 2.9% collagen; Panel C)**. Staining is consistent with patchy fibrosis patterns seen in older mdx mice

There was no significant collagen content/fibrosis found in BL10 hearts. Mdx hearts exposed to isoproterenol only for 2 weeks showed 1.4 ± 1.1% fibrosis and those hearts exposed to isoproterenol and treated with P188 showed 1.3 ± 1.1% fibrosis. After 4 weeks of isoproterenol exposure, mdx hearts showed 3.0 ± 2.8% and those hearts exposed to isoproterenol and treated with P188 showed 2.9 ± 2.1% fibrosis (Figure 3). Mdx control mice showed 1.1 ± 0.4% after 4 weeks, thus there was a significant increase in cardiac fibrosis in 4 week exposed and treated mdx groups (p < 0.05).

### Evans Blue Dye (EBD) positive cells

The number of EBD positive cells in the hearts of mdx mice showed that mice exposed to isoproterenol only had 5 ± 7 cells compared to 18 ± 15 cells per entire heart section in P188 treated isoproterenol exposed cells (p < 0.03). Untreated mdx hearts had no significant EBD staining at this age (12 weeks).

## Discussion

This study shows that chronic, intermittent P188 may be effective in protecting cardiac function in a subcutaneous low dose isoproterenol stress model over a 4 week period in dystrophin deficient hearts. Mdx mice treated with P188 during exposure to isoproterenol were able to maintain increased systolic function (46 ± 6 vs. 35 ± 6 SF%; p < 0.01) compared to untreated isoproterenol exposed mdx mice suggesting that chronic, intermittent injections of intraperitoneal P188 can stabilize cardiac muscle cell membranes *in vivo*. These results also provide a model for the development of mild cardiomyopathy and cardiac fibrosis in 2 month old mdx mice over a period of 4 weeks and allows for quicker screening of potential drugs targeting cardiac function and fibrosis.

We also showed that mdx mice remain exquisitely sensitive to *in vivo *cardiac stress from the beta agonist administration. All mdx mice died within 24 hours at doses of 30, 15, 10, 5 and 1 mg/kg/day and could only tolerate a six-fold decreased isoproterenol dose (0.5 mg/kg/day) compared to BL10 control mice. Even at this dosing, mdx mice had a shortening fraction of 42 ± 6% at 24 hours that decreased to 38 ± 5% at 2 weeks and 35 ± 6% at 4 weeks. P188 treatment did not improve survival at the higher levels of isoproterenol. This data suggests that the effectiveness to chronic intraperitoneal injections of P188 is limited and cannot stabilize cardiac cells as well as acute, intravenous administration.

P188 was previously studied in dystrophin deficient animal models and shown to prevent acute cardiac failure after dobutamine-induced cardiac stress in mdx mice. These studies utilized acute, intravenous administration [9]. Using chronic, intermittent dosing, our non-invasive *in vivo *cardiac stress model of subcutaneous isoproterenol administration and cardiac assessment using echocardiography demonstrated similar results. Yasuda et al. (2005) concluded that the primary effect of P188 was to acutely restore myocyte compliance and improve end-diastolic volumes. While we did not measure these parameters, increased systolic function that was equal to BL10 mice demonstrated an increased capacity to tolerate cardiac stress and is likely related to improved myocyte compliance. P188 can also decrease blood viscosity by stabilizing red blood cell membranes; decreasing red blood cell adhesion; and improving microvascular blood flow [19,20]. These changes can decrease effective afterload and are potential physiological mechanisms that could improve heart rates and cardiac systolic function in P188 treated mice.

Townsend et al. (2010) recently reported that a chronic 8 week infusion of P188 (60 mg/kg/h) in golden retriever muscular dystrophy (GRMD) dogs showed significantly decreased cardiac fibrosis and prevented ventricular dilation [21]. Our study did not show a significant decrease in cardiac fibrosis in P188 treated mice (3.0% vs. 2.9%). This may be related to our single daily dosing protocol that provided sufficient P188 to partially stabilize some cardiomyocytes and improve cardiac function, but not enough to prevent significant myocyte loss and fibrosis. The GRMD model also shows a more severe, naturally occurring cardiomyopathy which is difficult to replicate in the mouse model, even with isoproterenol exposure. Thus, fibrotic signaling pathways in the GRMD model were increased compared to the mdx mouse, and P188 therapy showed more significant modulation. The mdx mouse was so sensitive to isoproterenol stress that we were unable to test higher doses that could have increased fibrosis, without mortality, to better evaluate the anti-fibrotic effectiveness of P188.

Our study also did not show ventricular dilation, but rather displayed decreased left ventricular internal dimensions. This was secondary to increased left ventricular wall thickness and mass, seen in both P188 treated and untreated mice from the increased cardiac workload. Previous studies have shown both dilated and non-dilated cardiomyopathy in mdx mice [2,3]. In the presence of isoproterenol, untreated mdx mice were able to show cardiac hypertrophy similar to BL10 mice and this is likely related to compensatory mechanisms that are still present in the 3 month old mice that become decreased with age.

We unexpectedly demonstrated a significant increase in Evan's blue dye (EBD) positive fibers in P188 treated mdx hearts. Quilan et al. (2006) also found similar unexpected results studying skeletal muscles in exercised mdx mice, showing equal or increased %-EBD penetration in P188 treated rectus femoris muscle [22]. However, although we found an increase in EBD positive fibers, these hearts showed increased systolic function compared to untreated hearts. Based on these findings, we propose that P188 can stabilize mildly damaged cells, especially at lower effective doses. These cells remain functional but become EBD positive on histology. Also, there is the potential that P188 facilitates EBD entry into cells without compromising function.

We showed significantly lower blood pressures in mdx mice at baseline compared to controls. This was previously reported in 9 month old mdx mice [3]. P188 treatment had no effect on blood pressure levels, as was also seen in the study by Townsend et al. (2010) [21]. The mechanism for decreased blood pressures is not clear at this time and may be related to cardiac or vascular dysfunction, or both [23]. Previous studies demonstrated that the myocardium can generate more force after treatment with P188 [9,21]. This study also demonstrates improved cardiac function after P188 treatment. However, we do not see a corresponding effect in blood pressure. Thus, there is likely still an undetermined vascular component to decreased systolic blood pressures in mice that was not compensated for at this level of improved cardiac function. This supports previous literature that shows that mdx mice have abnormal vascular endothelium responses and blood pressure regulation [24,25].

This study did not show an improvement in skeletal muscle specific force using in vitro measurements. Ng et al. (2008) found that P188 addition to the muscle bathing solution reduced the force deficit in the isolated, whole lumbrical muscle [26]. Previous studies also showed that P188 restored compliance to isolated cardiac myocytes in solution, but these studies utilized acute administration of P188. When myocytes were used after a P188 washout period, properties returned to those of untreated animals [9,21]. We did not measure P188 serum levels and cannot estimate peak levels of exposure. While benefits were seen in cardiac function, no significant improvements in skeletal muscle function or cardiac fibrosis were seen in this study secondary to the chronic, intermittent dosing schedule. Thus, there is mounting evidence that P188 is most effective during acute administration in both cardiac and skeletal muscle.

## Conclusion

This study reports important pre-clinical data that chronic intraperitoneal administration of P188 to mdx mice stressed with subcutaneous isoproterenol can potentially improve cardiac systolic function. Previous studies support a role for acute treatment of P188 in stressed dystrophin deficient animal models [9,21]. These results support further study of daily P188 therapy in DMD patients. P188 could also prevent cardiac damage in times of acute stress, including orthopedic surgeries, respiratory failure and decompensated heart failure with a continuous, intravenous infusion. As advances in the treatment of DMD skeletal muscle pathology continue, it is as important to also develop cardiac muscle therapies so that DMD patients do not increasingly succumb to cardiac failure [27]. Based on these findings, P188 acute and chronic therapy may provide benefits for patients with Duchenne muscular dystrophy and further studies are encouraged.

## Competing interests

The authors declare that they have no competing interests.

## Authors' contributions

CFS participated in the study conception and design, acquisition of data, analysis and interpretation of data and drafted the manuscript. ADG was responsible for acquisition of data and analysis/interpretation of data. QY was responsible for acquisition of data and analysis/interpretation of data. AS was responsible for acquisition of data and analysis/interpretation of data. JHV was responsible for acquisition of data and analysis/interpretation of data. EPH was responsible for study conception and design and drafted the manuscript. KN was responsible for study conception and design, data analysis/interpretation and drafted the manuscript. All authors read and approved the final manuscript.

## Pre-publication history

The pre-publication history for this paper can be accessed here:

http://www.biomedcentral.com/1471-2261/11/20/prepub

## Supplementary Material

Additional file 1 ***In vitro***** muscle testing data for the extensor digitorum longus (EDL) in female mdx mice exposed to isoproterenol and treated with P188 over 2 and 4 weeks and compared with untreated female mdx mice of the same age**. There are no significant differences between groups. This file contains data obtained from *in vitro *muscle force testing of the EDL in female mdx mice exposed to isoproterenol and treated with P188 over 2 and 4 weeks and compared with untreated female mdx mice of the same age. Click here for file
