# Supplementary material for: Membrane Sealant Poloxamer P188 Protects Against Isoproterenol Induced Cardiomyopathy in Dystrophin Deficient Mice
Source: BMC Cardiovasc Disord. 2011 May 16;11:20. doi: 10.1186/1471-2261-11-20 (PMC3123649; doi:10.1186/1471-2261-11-20)
Supplement: Additional file 1 — In vitro muscle testing data for the extensor digitorum longus (EDL) in female mdx mice exposed to isoproterenol and treated with P188 over 2 and 4 weeks and compared with untreated female mdx mice of the same age. There are no significant differences between groups. This file contains data obtained from in vitro muscle force testing of the EDL in female mdx mice exposed to isoproterenol and treated with P188 over 2 and 4 weeks and compared with untreated female mdx mice of the same age. [file 1471-2261-11-20-S1.DOC]

Additional file 1: *In vitro* muscle testing data for the extensor digitorum longus (EDL) in female mdx mice exposed to isoproterenol and treated with P188 over 2 and 4 weeks and compared with untreated female mdx mice of the same age. There are no significant differences between groups.

| Mdx Group | Body mass (g) | EDL mass (mg) | CSA (mm^2^) | P_t_ (mN) | P_o_ (mN) | sP_o_ (kN/m^2^) |
| --- | --- | --- | --- | --- | --- | --- |
| Untreated control, n=3 | 24.6±1.0 | 11.8±0.7 | 1.9±0.1 | 94±10 | 297±35 | 157±14 |
| 2 wk iso,  n=5 | 25.4±1.5 | 11.8±1.7 | 1.9±0.3 | 82±19 | 268±62 | 142±24 |
| 2 wk iso/P188, n=5 | 26.4±0.9 | 12.7±0.7 | 2.1±0.2 | 97±17 | 337±31 | 161±17 |
| 4 wk iso,  n=5 | 24.3±1.4 | 11.4±1.8 | 1.8±0.3 | 98±13 | 351±36 | 198±35 |
| 4 wk iso/P188, n=5 | 23.6±1.2 | 11.8±1.5 | 1.9±0.2 | 97±12 | 327±40 | 171±26 |

Iso – isoproterenol; wk – week; g – grams; mg – milligrams; CSA – cross sectional area; P_t_ – twitch force; P_o_ – maximal force; sP_o_ – specific force; mN – millinewtons; kN – kilonewtons; m^2^ – meter squared; mm^2^ – millimeter squared; EDL – extensor digitorum longus
